# Quantitative Proteomics of Polarised Macrophages Derived from Induced Pluripotent Stem Cells

**DOI:** 10.3390/biomedicines10020239

**Published:** 2022-01-23

**Authors:** Gavuthami Murugesan, Lindsay Davidson, Linda Jannetti, Paul R. Crocker, Bernd Weigle

**Affiliations:** 1 Division of Cell Signalling and Immunology, School of Life Sciences, University of Dundee, Dundee DD1 5EH, UK; g.z.murugesan@dundee.ac.uk (G.M.); p.r.crocker@dundee.ac.uk (P.R.C.); 2 Human Pluripotent Stem Cell Facility, School of Life Sciences, University of Dundee, Dow Street, Dundee DD1 5EH, UK; l.u.davidson@dundee.ac.uk; 3 Division of Cancer Immunology and Immune Modulation, Boehringer Ingelheim Pharma GmbH & Co. KG, 88397 Biberach an der Riss, Germany; linda.jannetti@boehringer-ingelheim.com

**Keywords:** iPSC, macrophages, polarisation, secretome, proteomics

## Abstract

Macrophages (M_Φ_) are highly heterogenous and versatile innate immune cells involved in homeostatic and immune responses. Activated M_Φ_ can exist in two extreme phenotypes: pro-inflammatory (M1) M_Φ_ and anti-inflammatory (M2) M_Φ_. These phenotypes can be recapitulated in vitro by using ligands of toll-like receptors (TLRs) and cytokines such as IFNγ and IL-4. In recent years, human induced pluripotent stem cells (iPSC)-derived M_Φ_ have gained major attention, as they are functionally similar to human monocyte-derived M_Φ_ and are receptive to genome editing. In this study, we polarised iPSC-derived M_Φ_ to M1 or M2 and analysed their proteome and secretome profiles using quantitative proteomics. These comprehensive proteomic data sets provide new insights into functions of polarised M_Φ_.

## 1. Introduction

Macrophages (M_Φ_) are innate immune cells that have gained increasing attention in basic and biopharmaceutical research. They play crucial roles not only in homeostasis, immune regulation, and pathogen defence, but also in the pathogenesis of an increasing number of diseases. M_Φ_ display remarkable plasticity and can exist in a broad spectrum of activation states in response to a plethora of internal and external stimuli. At the extreme ends of the spectrum are the ‘classically activated’ or proinflammatory (M1) M_Φ_ and the ‘alternatively activated’ or anti-inflammatory (M2) M_Φ_, as described in the literature [1].

For human M_Φ_ research, peripheral blood monocyte-derived M_Φ_ (MDM) and monocytic cell lines are commonly used. However, there are limitations to the use of MDM due to donor-to-donor variability and restricted availability of donors. Also, M_Φ_ derived from transformed monocytic cell lines are not able to completely recapitulate all aspects of M_Φ_ physiology, as they exhibit limited plasticity and often carry unknown mutations [2,3]. More recently, iPSC-derived M_Φ_ (iPSDM) have emerged as a valuable platform for the generation of unlimited numbers of M_Φ_ that are functionally similar to MDM [4]. In addition, the genetic manipulation of iPSCs to knock out genes of interest has paved the way for a better understanding of M_Φ_ biology and has provided a platform for testing drug concepts that interfere with M_Φ_ function [5,6].

There are extensive transcriptomic data available that have provided valuable insights on the expression profiles of polarised M_Φ_ and of specific cell surface markers. However, mRNA levels do not always correlate with protein abundance due to post-transcriptional processing, mRNA stability, and post-translational modifications. In addition, the M_Φ_ secretome cannot be fully described by transcriptomics. Comprehensive proteomics is a powerful approach to gain important functional insights into the M_Φ_ immune response to different stimuli [7]. Therefore, we determined changes in both cellular (proteome) and extracellular (secretome) responses of differentially polarised iPSDM using label-free quantitative proteomics. The resting M_Φ_ (M0) that were polarised using toll-like receptor (TLR) ligand, lipopolysaccharide (LPS), and interferon gamma (IFNγ) are referred to as M1, while M_Φ_ polarised using interleukin-4 (IL-4) are referred to as M2, for simplification [8]. We demonstrate that ~5% of proteins are expressed differentially between M1 and M2 and show that, by principal component analysis, M1 are more distinct from M0 than M2 are from M0. In addition, novel cell surface proteins preferentially expressed on polarized iPSDM were identified that had not previously been associated with M_Φ_ polarisation. In order to investigate their potential as polarisation markers, their expression was validated by FACS using iPSDM and donor-derived MDM. Furthermore, our secretome analyses identified a large number of proteins released from differentially polarised iPSDM.

## 2. Materials and Methods

### 2.1. Antibodies and Reagents

RPMI medium, DPBS without Ca^2+^ and Mg^2+^, penicillin-streptomycin, Glutamax, microplate BCA protein assay, trypsin-EDTA, and TEAB were from Thermo Scientific, Waltham, MA, USA; trypsin protease-MS grade, sodium dodecyl sulphate, acetonitrile, HPLC-grade ethanol, and water were from Sigma, Dorset, UK; human Fc block and Alexa Fluor^®^ anti-human CD300A Cat# 566342 were from BD Bioscience, Berkshire, UK; PE anti-human CD68 Cat# 130-118-486 was from Miltenyi Biotec, Surrey, UK; PE anti-human CD109 Cat# 323305, APC anti-human LILRB2 Cat# 338707, APC anti-human Siglec-10 Cat# 347606, APC anti-human CD206 Cat# 321109, APC anti-human CD80 Cat# 305219, APC anti-human CD86 Cat# 374208, PE/Cy7 anti-human CD14 Cat# 367112, and recombinant human M-CSF were from Biolegend, San Diego, CA, USA. X-VIVO15 Serum free medium Cat# BE02-060Q was from Lonza, Basel, Switzerland. Recombinant human BMP4 (Cat# 120-05), SCF (Cat# 300-07), VEGF (Cat# 100-20) and IL-3 (Cat# 200-03) were from Peprotech, London, UK. EmbryoMax^®^ 0.1% Gelatin solution was from Millipore, London, UK. Y-27632 (Cat# 1254/10) was from Bio-Techne, Abingdon, UK.

### 2.2. Differentiation of iPSC Cells to M_Φ_

Wibj2 is an iPSC line established from fibroblasts of a 55-years old female [9]. These cells were provided by the HipSci consortium (www.hipsci.org, accessed on 27 November 2021) and maintained and quality controlled by the Human Pluripotent Stem Cell Facility (University of Dundee). The monocytic lineage differentiation protocol was adapted from Wilgenburg et al. [10] and Lopez-Yrigoyen et al. [11]. Briefly, Wibj2 iPSC cells were cultured in mTESR medium. At day 0, embryoid bodies (EBs) were generated by seeding 1 × 10^4^ cells per well in a 96-well Ultralow attachment V-bottomed wells in mTESR medium supplemented with SCF (20 ng/mL), VEGF (50 ng/mL), BMP4 (50 ng/mL), and Y-27632 (10 µM). The plate was centrifuged at 300× *g* for 5 min at room temperature. On day 4, EBs were harvested and seeded at 10 to 15 EBs per well in 0.1% gelatin-coated 6-well plates. EBs were cultured in X-VIVO15 medium supplemented with IL-3 (25 ng/mL) and M-CSF (100 ng/mL). The medium was changed every 3 to 4 days. After 21 days, monocyte-like suspension cells were harvested from the supernatant every 3 to 4 days. The monocytes were differentiated to M_Φ_ in X-VIVO15 supplemented with M-CSF (100 ng/mL) for 7 days in non-TC treated plates.

### 2.3. Peripheral Blood Monocyte-Derived M_Φ_

Human buffy coats were from the Scottish Blood Transfusion Service, Edinburgh, UK. Isolation of peripheral blood mononuclear cells (PBMCs) from buffy coats was performed using density gradient centrifugation according to the manufacturer’s protocol (Lymphoprep, STEMCELL Technologies, Cambridge, UK). For each donor, 50 × 10^6^ PBMCs were used to isolate CD14+ monocytes using the EasySepTM human monocyte isolation kit (STEMCELL Technologies, Cambridge, UK; Cat# 19359) according to the manufacturer’s instructions. Monocytes were differentiated using X-VIVO15 medium containing 100 U/mL Penicillin-Streptomycin and 1X GlutaMAX supplemented with M-CSF (100 ng/mL).

### 2.4. Phagocytosis Assay

iPSDM (1 × 10^6^ cells per well) were washed twice with PBS and RPMI medium without serum was added. Fluorescein (FITC)-conjugated Zymosan A particles (Cat# Z2841, ThermoScientific) (5 particles per cell) were added and phagocytosis was allowed to occur at 37 °C for 1 h. Cells were then washed with PBS followed by trypan blue (250 µg/mL) containing PBS to neutralise all the cell-bound Zymosan particles. Cells were then detached using PBS containing 2 mM EDTA and were analysed by flow cytometry.

### 2.5. Flow Cytometry

iPSC-derived M_Φ_ and peripheral blood monocyte-derived M_Φ_ were harvested using PBS containing 2 mM EDTA. Monocytes and M_Φ_ were stained for viability using DAPI or Zombie Aqua viability dye (Biolegend) for 30 min at 4 °C. Cells were then Fc receptor blocked for 20 min at 4 °C using the human BD Fc block and stained with fluorophore-conjugated antibodies for 30 min at 4 °C. Following staining, cells were washed in FACS buffer (2% BSA in PBS) and analysed by using BD FACSCanto II flow cytometer and FlowJo (Version 10.7.1, FlowJo LLC, Ashland, OR, USA).

### 2.6. Processing of Proteome and Secretome Samples

Proteomic samples were processed by using the SP3 protocol [12]. Briefly, 1 × 10^6^ iPSDM per well were stimulated with IFNγ (50 ng/mL) + LPS (10 ng/mL) (M1 M_Φ_) or IL-4 (20 ng/mL) (M2 M_Φ_) for 48 h. Cells were washed twice with PBS and lysed in buffer containing 4% SDS, 10 mM TCEP and 50 mM TEAB.

For secretome analysis, iPSDM (1 × 10^6^ per well) were polarised to M1 and M2 for 24 h. Cells were then washed twice with PBS, media were changed to RPMI without phenol red and FBS (Cat# 32404014, Thermo Scientific, Waltham, UK) was supplemented with 100 ng/mL M-CSF and incubated at 37 °C. After 14 h, culture supernatants were harvested, passed through 0.45 µm filters to remove any cell debris, and concentrated 10 times using Amicon centrifugal filters (Cat# UFC801024, Millipore, London, UK).

Cell lysates/culture supernatants were boiled for 5 min and sonicated (15 cycles, 30 s on/30 s off). Protein concentrations were determined using a MicroBCA assay kit and equal amounts of lysates were taken for further processing. The samples were alkylated using iodoacetamide (20 mM) and incubated at room temperature in the dark for 1 h. To the alkylated lysates, SP3 beads were added to a final concentration of 0.5 µg/µL of processing volume, followed by an equal volume of ethanol (1:1 ethanol: cell lysate). The mixture was incubated at 24 °C for 5 min at 1000 rpm and the tubes were placed on the magnetic rack until the beads settled onto the tube wall. The unbound supernatant was discarded and the beads were rinsed thrice in 80% ethanol while on the magnetic rack. After removing all the residual ethanol, the beads were air-dried and resuspended in a digestion solution (100 mM ammonium bicarbonate) containing LysC and trypsin (at 1:25 *wt*/*wt* of protein: proteases) and sonicated in a water bath. The bead mixture was incubated at 37 °C at 1000 rpm overnight. After the protease digestion was complete, the bead mixture was centrifuged at 20,000× *g* for 1 min. The tubes were placed on the magnetic rack and the supernatants were transferred to fresh tubes. The supernatants were then dried and suspended in 50 µL of 1% formic acid prior to analysis with LC-MS.

### 2.7. LC-MS/MS Analysis

LC-MS analysis was performed by the FingerPrints Proteomics Facility (University of Dundee). Analysis of peptide readout was performed on a Q Exactive^™^ plus, Mass Spectrometer (Thermo Scientific) coupled to a Dionex Ultimate 3000 RS (Thermo Scientific). LC buffers used were the following: buffer A (0.1% formic acid in Milli-Q water (*v*/*v*)) and buffer B (80% acetonitrile and 0.1% formic acid in Milli-Q water (*v*/*v*). An equivalent of 0.75 µg of each sample were loaded at 10 μL/min onto a trapping column (100 μm × 2 cm, PepMap nanoViper C18 column, 5 μm, 100 Å, Thermo Scientific) equilibrated in 0.1% TFA. The trap column was washed for 3 min at the same flow rate with 0.1% TFA and then switched in-line with a Thermo Scientific, resolving C18 column (75 μm × 50 cm, PepMap RSLC C18 column, 2 μm, 100 Å). The peptides were eluted from the column at a constant flow rate of 300 μL/min with a linear gradient from 2% buffer B to 5% buffer B in 5 min, then from 5% buffer B to 35% buffer B in 125 min, and then to 98% buffer B within 2 min. The column was then washed with 98% buffer B for 20 min and equilibrated for 17 min with 2% Buffer B The column was kept at a constant temperature of 50 °C. Q-exactive plus was operated in data dependent positive ionization mode. The source voltage was set to 2.7 Kv and the capillary temperature was 250 °C.

A scan cycle comprised MS1 scan (*m*/*z* range from 350–1600, ion injection time of 20 ms, resolution 70,000 and automatic gain control (AGC) 1 × 10^6^) acquired in profile mode, followed by 15 sequential dependent MS2 scans (resolution 17500) of the most intense ions fulfilling predefined selection criteria (AGC 2 × 10^5^, maximum ion injection time 100 ms, isolation window of 1.4 *m*/*z*, fixed first mass of 100 *m*/*z*, spectrum data type: centroid, intensity threshold 2 × 10^4^, exclusion of unassigned, singly and >7 charged precursors, peptide match preferred, exclude isotopes on, dynamic exclusion time 45 s). The HCD collision energy was set to 27% of the normalized collision energy. Mass accuracy was checked before the start of samples analysis.

### 2.8. Quantification of Proteome and Secretome Data

The raw mass spectrometric data were analysed using MaxQuant software (version 1.5.5.1) [13] and the Andromeda search engine [14]. The false discovery rate (FDR) was set to 1% for both peptides and proteins. MaxQuant scored peptide identifications based on a search with a permissible mass tolerance of 7 ppm for precursor ions and 0.5 Da for fragment ions. The enzyme specificity was set to LysC/Trypsin. Other parameters used were the following: fixed modifications, cysteine carbamidomethylation; variable modifications, deamidation, protein *N*-acetylation and methionine oxidation; missed cleavages, 2; and minimum peptide length was set to 7. The Andromeda search engine was used to match MS/MS data against the human Uniprot database. The ‘match between runs’ feature was activated to transfer identification information to other LC-MS/MS runs based on ion masses and retention times. Minimum peptide ratio count was set to 2 and the relative quantitation between the peptides identified across different conditions was based on LFQ and iBAQ intensities. The normalised peptides intensities across samples were downloaded as an output file ’proteingroups.txt’.

### 2.9. Analysis of Proteomic Data

Perseus software version 1.6.7.0 was used for statistical analyses of the proteomic data. All the potential contaminants, reverse peptides, and peptides ‘only identified by site’ were removed from the data and the absolute copy number per cell and concentration were estimated by using a Proteomic ruler [15]. Proteins (reproducible in all biological replicates) with log intensities of copy number per cell and concentrations greater than 2–fold and *p*-value greater than 0.05 were considered differentially regulated across polarised M_Φ_.

Gene Ontology (GO) over representation enrichment analysis was done by using the WEB-based GEne SeT AnaLysis Toolkit [16]. Heat maps of estimated protein concentrations were generated using Broad Institute software Morpheus (https://software.broadinstitute.org/morpheus, accessed on 27 November 2021).

### 2.10. Graphs and Statistics

All the graphs (mean ± SEM) were plotted using GraphPad Prism version 9. Statistical analyses were determined using ANOVA followed by Tukey’s post hoc HSD test for pairwise comparison; *p*-values <0.05 were considered significant.

## 3. Results and Discussion

### 3.1. Characterisation of iPSC-Derived M_Φ_

**Figure 1:**
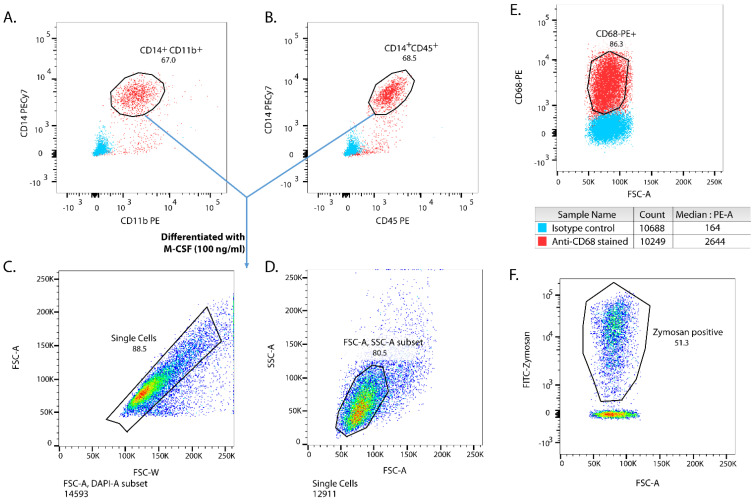
Induced pluripotent stem cell (iPSC)-derived monocytes and macrophages (M_Φ_). iPSC-derived monocytes were tested for the expression of monocyte markers, such as CD14, CD11b (**A**), and CD45 (**B**). iPSC derived monocytes were differentiated into macrophages in X-VIVO15 medium supplemented with M-CSF (100 ng/mL) for 7 days. Gating strategy for iPSC derived-M_Φ_ (iPSDM) is shown (**C**,**D**). iPSDM were stained for the macrophage marker CD68 (**E**) and tested for their phagocytic activity using fluorescein conjugated Zymosan particles (**F**).

Wibj2 iPSCs were differentiated to monocytes using a protocol adapted from Wilgenburg et al. [10] and Lopez-Yrigoyen et al. [11] as described in the Materials and Methods section. The monocyte yield was assessed by using monocyte markers, such as CD14 and CD11b (Figure 1A), and a pan-leukocyte marker, CD45 (Figure 1B). Monocyte yield was around 65 to 80% (Figure 1A,B) which is in line with Lopez-Yrigoyen et al. [11]. The monocytes were further differentiated into M_Φ_ using X-VIVO15 medium supplemented with M-CSF for 7 days in non-TC treated plates, with media change at day 4 to remove other contaminating cells (Figure 1C,D). The adherent M0 iPSDM were CD68-positive (Figure 1E). Since phagocytosis of pathogens or cell debris is one of the hallmark functions of M_Φ_, we investigated the phagocytic activity of M0 iPSDM. After incubation of cells with FITC-Zymosan particles for 30 min, ~50% of iPSDM were Zymosan-positive (Figure 1F), confirming functional similarity with MDM [17].

**Figure 2:**
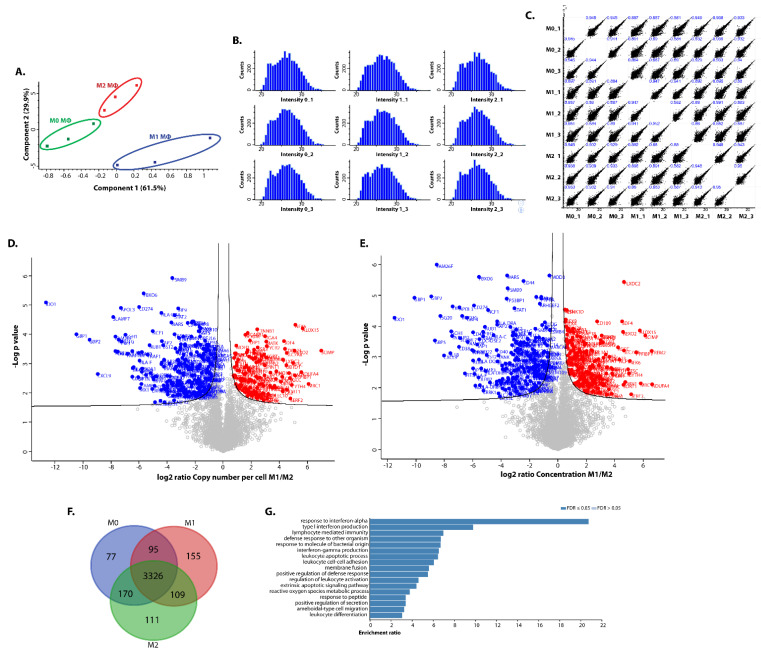
Proteomic analyses of differentially polarised iPSDM. (**A**) Principal component analysis scores plot showing clustering of iPSDM according to their polarisation. (**B**) Histograms displaying the distribution of log2-transformed intensities of samples. (**C**) Scatter plots of protein copy numbers per cell show high correlation of biological replicates (Pearson’s coefficient close to 1). (**D**) Volcano plot showing fold changes in proteins using copy number vs. log *p*-values from mass spectrometry of M1 and M2 macrophages. (**E**) Volcano plot showing changes in concentrations vs. log *p*-values. (**F**) Venn diagram showing the number of proteins differentially expressed by polarised iPSDM. (**G**) Gene ontology analysis of the protein hits enriched in M1 iPSDM.

To address whether plasticity of iPSDM is similar to human MDM, we polarised M0 iPSDM to M1- or M2-phenotypes using IFNγ plus LPS or IL-4, respectively. These samples were processed and analysed by one shot quantitative label-free mass spectrometry (MS). More than 4000 proteins were identified and copy number per cell was estimated using the ‘proteomic ruler’ method [15]. Principal component analysis (PCA) of the proteins identified by MS showed that M0 and M2 iPSDM are more closely related to each other compared to M1 iPSDM (Figure 2A). These findings are in line with published transcriptomics and nCounter gene expression analyses of polarised MDM [18,19]. In addition, it has been shown previously [20] that M-CSF alone polarises M_Φ_ more to a M2-like phenotype. Histograms of the log-transformed intensities follow normal distributions, which suggests that the data are of good quality (Figure 2B). Moreover, variability within the replicates was from 0.89 to 0.96, indicating good reproducibility of MS runs (Figure 2C). Volcano plots were generated by plotting the *p*-value against the M1:M2 ratio of log-transformed copy numbers per cell (Figure 2D) and several established marker proteins for polarized M_Φ_, such as IDO1, CXCL9, ALOX15, and CD206, became immediately apparent. To normalise for the effect of polarisation on cell size, another volcano plot was generated using the log-transformed values of concentration (in nanomolar) (Figure 2E). When considering ‘differentially regulated’ proteins with a fold change of ≥2 and *p*-value ≤ 0.05 in the volcano plots, many proteins were found to be shared between resting and polarised iPSDM, while only ~5% of proteins were differentially regulated (Figure 2F and Table S1). As reflected in the Venn diagram, fewer proteins (220) were differentially regulated in M2 vs. M0 iPSDM as compared to the number of proteins (264) differentially expressed in M1 vs. M0 iPSDM, corroborating results from transcriptomics analyses of MDM (Figure 2F) [18]. In line with our expectation, GO analysis of the protein IDs upregulated in M1 iPSDM were found to be enriched in interferon and immune signalling (Figure 2G).

### 3.2. Polarisation-Induced Changes in the Expression of Membrane Proteins

**Figure 3:**
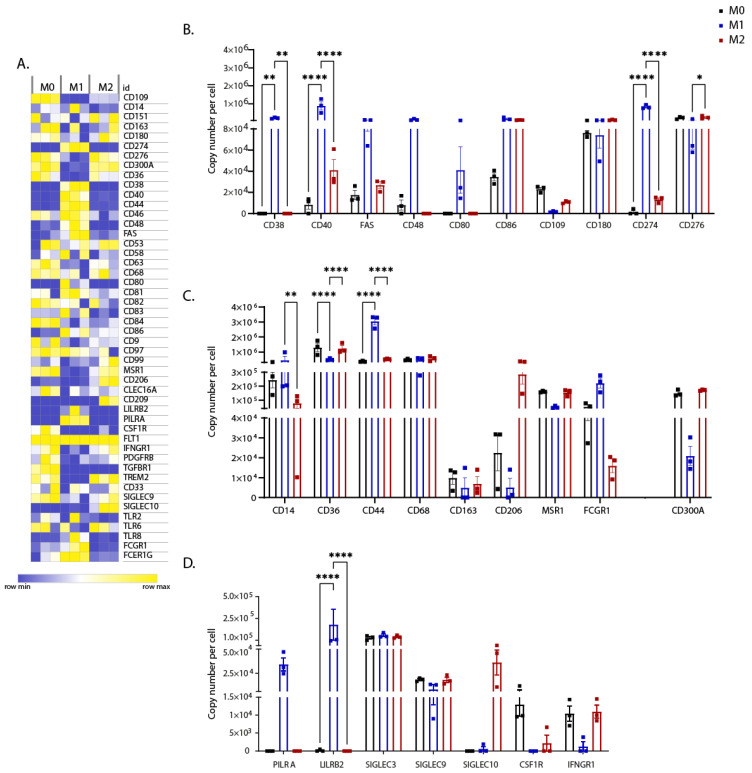
Expression levels of CD markers and other membrane proteins. (**A**) Heat map showing concentrations (in nM) of membrane proteins across biological replicates (n = 3) of differentially polarised iPSDM. (**B**–**D**) Graphs show the estimated copy numbers per cell using the proteomic ruler approach. *p* values: * 0.05, ** 0.01, **** 0.0001.

Proteomic data showed that many membrane proteins were differentially expressed in polarised iPSDM. We observed a good correlation between the changes in concentrations and copy numbers per cell (Figure 3A–D).

As expected, well-characterised markers such as CD48, CD80, and CD86 were highly expressed in M1 iPSDM (Figure 3A,B and Figure S1). These membrane proteins function as costimulatory molecules for T cell activation and effector function [21,22].

TNF receptor superfamily members such as CD40 and FAS were upregulated in M1 iPSDM (Figure 3A,B). Ligation of these receptors has been reported to induce secretion of pro-inflammatory cytokines and trigger apoptosis [23,24].

CD38 is a multifunctional exoenzyme that has been shown to promote secretion of pro-inflammatory cytokines in M_Φ_ [25]. As shown previously [26], IFNγ and LPS strongly induced expression of CD38 in iPSDM (Figure 3A,B).

**Figure 4:**
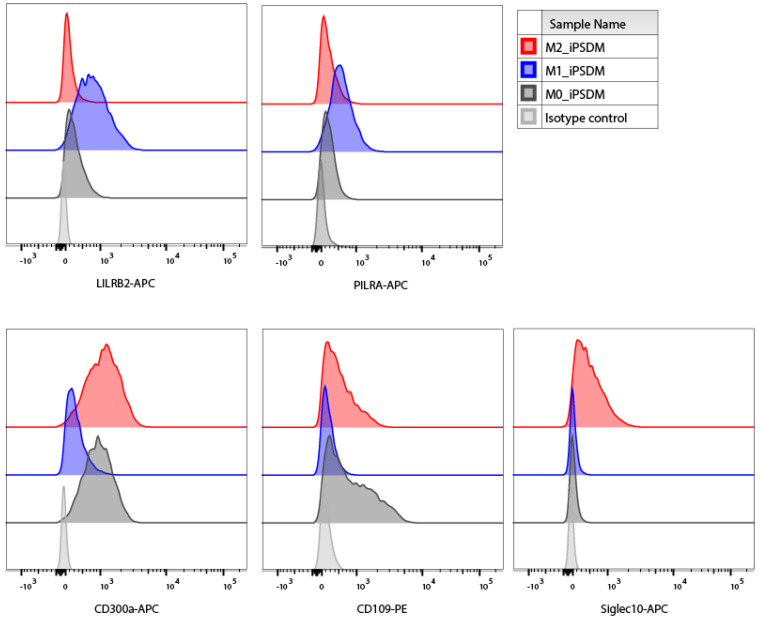
Expression of novel polarisation markers in iPSDM. iPSC-derived monocytes were differentiated to M_Φ_ for 7 days and polarised with IFNγ + LPS or IL-4 for 48 h. Polarised iPSDM were Fc blocked, stained for LILRB2, PILRA, CD300a, CD109, and Siglec-10 and analysed by FACS Canto. Representative of four independent experiments.

**Figure 5:**
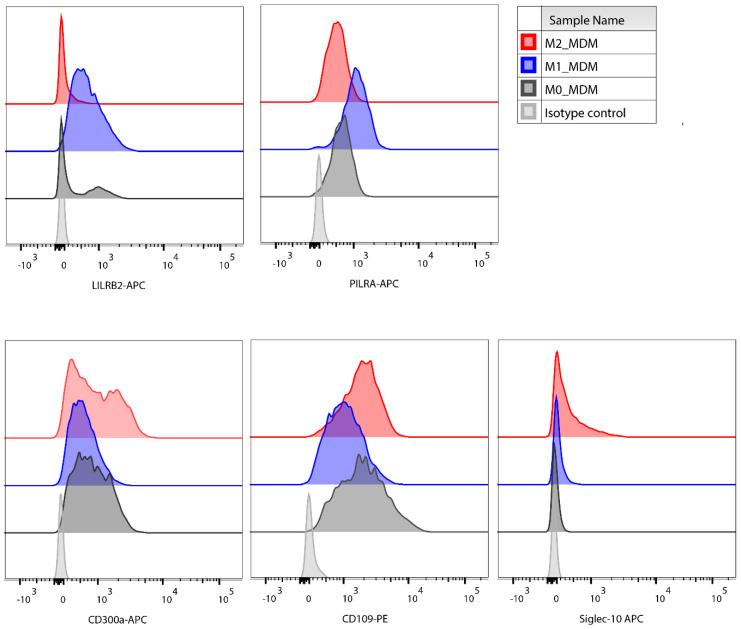
Validation of expression of novel polarisation markers in monocyte-derived macrophages (MDM). Peripheral blood monocyte-derived monocytes were differentiated to M_Φ_ for 7 days and polarised with IFNγ + LPS or IL-4 for 48 h. Polarised MDM were stained for LILRB2, PILRA, CD300a, CD109, and Siglec-10 and analysed by FACS Canto. Representative of four independent donors.

Proteomic analysis identified substantial downregulation of CD109 expression in M1 iPSDM (Figure 3A,B). FACS staining of M1 iPSDM and MDM confirmed low expression of CD109, a molecule that was initially identified as a coreceptor for TGFβ and has a role in inhibiting intracellular TGFβ signalling [27] (Figure 4 and Figure 5).

PD1/PD-L1 (CD274) is an immune checkpoint that dampens immune response mediated by M_Φ_ [28]. As shown previously [29,30,31], stimulation with IFNγ and LPS increased expression of CD274 in iPSDM (Figure 3A,B), which could be a mechanism of feedback inhibition of inflammation in M_Φ_.

Phagocytosis of pathogens and apoptotic cells is mediated by several receptors such as CD14, CD36, CD44, FCGR1 (CD64), MSR1, CD206, and CD163, all of which were highly expressed in M0 iPSDM (Figure 3A,C and Figure S1). We observed a significant increase in the expression of CD14, FCGR1, and CD44 in M1 iPSDM (Figure 3A,C). As shown previously for MDM [32], the well-characterised marker CD206 was upregulated in iPSDM on stimulation with IL-4 (Figure 3A,C).

CD300a is an inhibitor of phagocytosis of apoptotic cells in M_Φ_ [33]. Stimulation with IFNγ and LPS markedly reduced expression of CD300a in M1 iPSDM (Figure 3A,C) and this was validated by flow cytometry (Figure 4). We also observed similar changes in CD300a expression in polarized MDM, suggesting that this represents a physiologically important response (Figure 5 and Figure S3). To the best of our knowledge, this is the first demonstration that CD300a is regulated on polarized MF and may have important implications for its phagocytic functions.

Furthermore, in the M1 proteome, we observed enrichment of immune-inhibitory receptors, such as PILRA and LILRB2 (Figure 3A,D). This is in line with the previous reports demonstrating upregulation in mRNA levels of LILRB2 and PILRA on stimulation with LPS [34,35]. Most Siglecs (sialic acid binding Ig-like lectins) have an immunoinhibitory function. Our analysis showed that IL-4 induced Siglec-10 expression in iPSDM (Figure 3A,D), consistent with previous studies [36,37]. However, polarisation had little or no impact on the expression of Siglecs-3 and -9. The differential expression of these proteins in polarised human M_Φ_ was confirmed by FACS staining of both MDM and iPSDM (Figure 4, Figure 5 and Figure S3). Of note, the expression of LILRB2 and PILRA was highly restricted to M1 M_Φ_ by quantitative proteomics and FACS analyses, suggesting that these receptors are suitable markers for classically activated M_Φ_. However, we observed donor-to-donor variations in the expression levels of these novel markers in polarised MDMs (Figure S3).

Further proteomic analysis identified high expression of growth factor receptors such as CSF1R, PDGFR and TGFBR in M0 M_Φ_. However, polarisation of iPSDM with IFNγ and LPS downregulated the expression of these receptors (Figure 3A,D). As shown previously [38], stimulation with IFNγ significantly reduced the expression of its counter receptor IFNGR1 and this could be a feedback mechanism to dampen the inflammatory response in M_Φ_.

**Figure 6:**
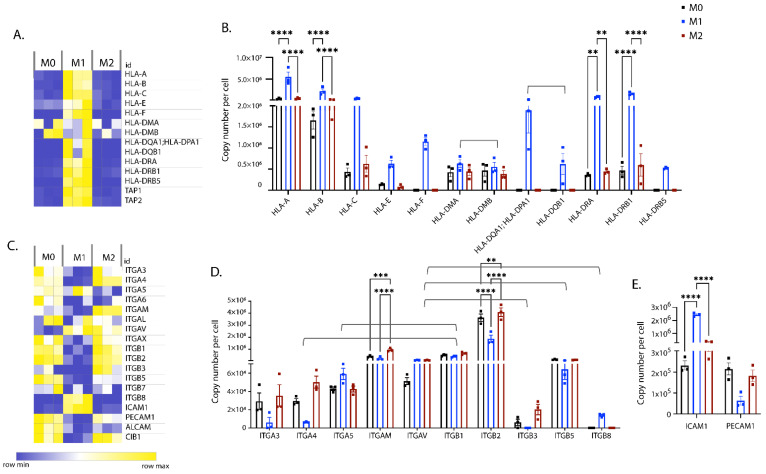
Expression levels of MHC and cell adhesion molecules. Heat map showing concentrations (in nM) of HLAs (**A**) and adhesion molecules (**C**) across biological replicates of differentially polarised iPSDM. Graphs show the estimated copy numbers per cell of HLAs (**B**), integrins (**D**) and cell adhesion molecules (CAMs) (**E**) using a Proteomic ruler. *p* values: ** 0.01, *** 0.001, **** 0.0001.

Another key function of M_Φ_ is the processing and presentation of antigens to other immune cells, via MHC molecules. MHC molecules are heterodimeric cell surface glycoproteins and are classified into two groups (MHC-I and -II), depending on the expression pattern and the type of peptides presented. Consistent with previous studies, the expression of MHC-I (HLA-A, -B, -C, -E and -F) and MHC-II (HLA-DR -DM, -DP and -DQ) were upregulated in M1 iPSDM (Figure 6A,B).

Integrins are a large family of heterodimeric transmembrane receptors that mediate phagocytosis, adhesion, and extravasation of M_Φ_ to the sites of inflammation. We observed an increase in expression of leukocyte integrins, such as CD11b (ITGAM α chain, ITGB2 β chain), and VLA4 (ITGA4 α chain, ITGB1 β chain), in M2 iPSDM (Figure 6C,D). While the majority of α and β-chains were downregulated in M1 iPSDM, we observed enrichment of ITGA5 (α5-chain), which pairs with ITGB1 (β1-chain), and ITGB8 (β8-chain), which pairs with ITGAV (αV-chain) (Figure 6C,D).

We next analysed the expression of adhesion molecules. As shown previously [39,40], ICAM was found to be enriched in the M1 proteome. In contrast, stimulation with IFNγ and LPS downregulated PECAM expression in iPSDM (Figure 6C,E). PECAM has been reported to negatively regulate LPS/TLR4 signalling, possibly through interaction with its ligand CD38 [41].

### 3.3. Expression of Interferon-Regulated Genes

**Figure 7:**
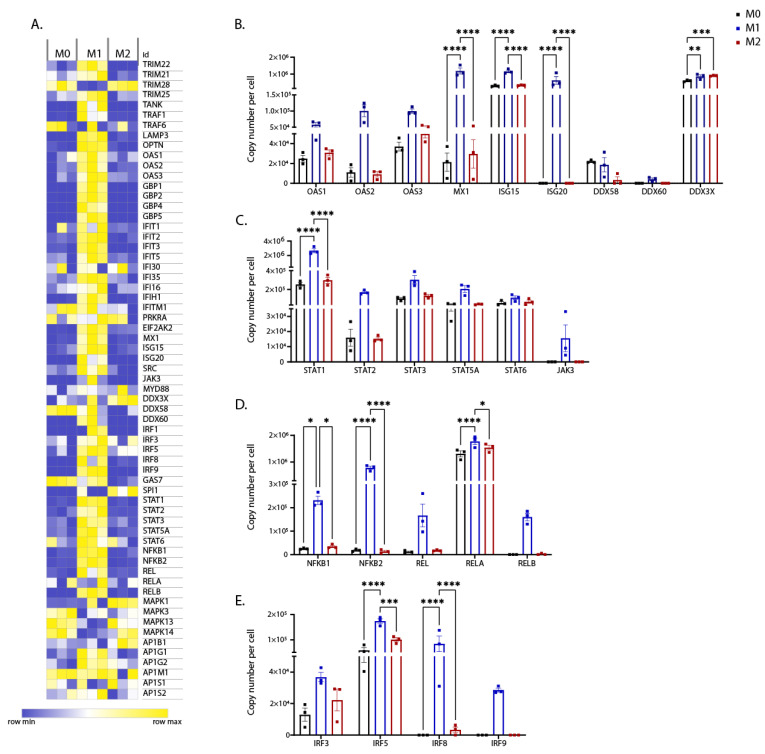
Differential expression levels of proteins involved in interferon (IFN) signalling. (**A**) Heat map showing differential expression of IFN regulated genes (concentrations in nM) across biological replicates of differentially polarised iPSDM. (**B**–**E**) Graphs show the estimated copy numbers per cell using the proteomic ruler method. *p* values: * 0.05, ** 0.01, *** 0.001, **** 0.0001.

IFNγ and IL-4 bind their cognate receptors and signal through kinases, such as JAKs, resulting in subsequent activation of transcription factors, such as STATs. Our proteomic analyses showed that JAK3, STAT1-4, and STAT5A were enriched in M1 proteome (Figure 7A,C). However, polarisation had little impact on STAT6 expression (Figure 7A,C). Phosphorylated STATs translocate to the nucleus, wherein they bind interferon regulatory factors (IRF) and induce transcription of IFN-regulated genes (IRGs). Interestingly, IRF3, 5, 8, and 9 were highly expressed in M1 iPSDM (Figure 7A,E). Furthermore, we observed enrichment of IRGs, such as ISG15, ISG20 IFIT1, IFIT2, IFIT2, IFIT5, IFITM, IFIH1, MX1, EIF2AK2, DDX60, OAS1, OAS2, OAS3, TRIM22, and TRIM25, in the M1 proteome (Figure 7A,B). Similarly, IFN-induced GTPases, such as GBP1, 2, 4, and 5, were highly expressed in M1 iPSDM (Figure 7A).

Next, we assessed the expression of the components of LPS/TLR4 signalling. NFĸB is the key transcription factor that mediates expression of inflammatory cytokines such as TNFα, IL12, and IL1B [42]. The NFĸB family of transcription factors includes RelA/p65, RelB, c-Rel, NFĸB1/p50, and NFĸB2/p52. Interestingly, all the NFĸB subunits were upregulated in M1 iPSDM (Figure 7A,D). However, there was no impact of polarisation on the expression levels of other downstream effectors of TLR4 signalling, such as MyD88, MAPK, TRAF6, and AP1 (Figure 7A).

Furthermore, we observed that the transcription factor SPI1 was enriched in M2 (IL-4) iPSDM (Figure 7A). SPI1 has been reported to be a crucial regulator of M2 polarisation in M_Φ_ [43].

Consistent with previous transcriptomic studies [44], the proteins involved in vesicle trafficking such as OPTN (optineurin) and LAMP3 were upregulated in M1 iPSDM (Figure 7A).

Taken together, our analyses suggest that the mediators of IFNγ and NFkB signalling are enriched in M1 M_Φ_.

### 3.4. Differential Expression of Proteins Involved in Metabolism

**Figure 8:**
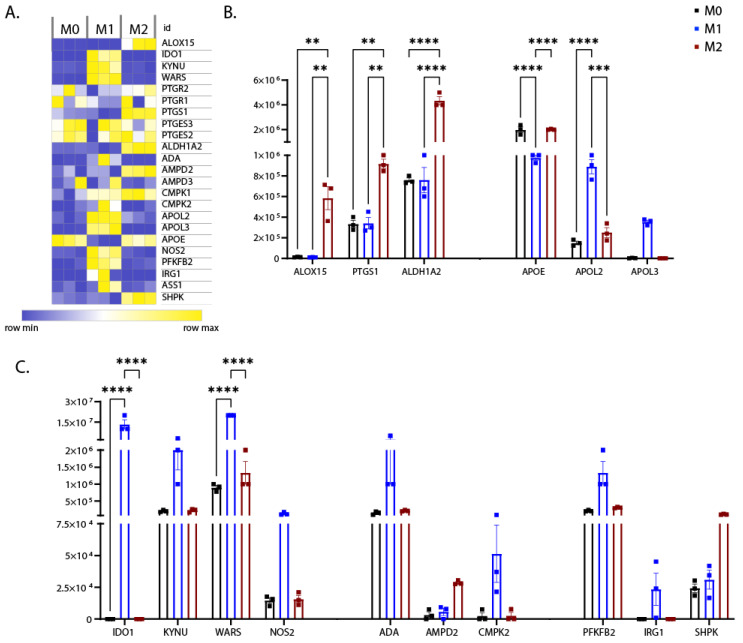
Expression levels of metabolic pathway proteins. (**A**) Heat map showing differential expression of proteins involved in metabolic pathways across biological replicates of differentially polarised iPSDM. (**B**,**C**) Graph shows the estimated copy numbers per cell using the proteomic ruler approach. *p* values: ** 0.01, *** 0.001, **** 0.0001.

Important mediators of inflammation resolution are derived from metabolism of prostaglandin, arachidonic acid, and retinoic acid (RA). Proteins implicated in metabolism of prostaglandin, such as PTGS1 [45] and arachidonic acid such as ALOX15 [46], were found to be enriched in the M2 proteome (Figure 8A,B). In addition, retinol dehydrogenase (ALDH1A2), the rate-limiting enzyme for RA biosynthesis [47,48], was also highly expressed in M2 iPSDM (Figure 8A,B).

Apolipoproteins are involved in the regulation of cholesterol levels and also function as effectors of the immune response to pathogens [49]. In the M1 proteome, we found enrichment of APOL2 and APOL3 (Figure 8A,B), in line with previous studies [50]. Furthermore, APOE, which has immunomodulatory function [51,52], was downregulated in M1 iPSDM (Figure 8A,B).

Tryptophan (Trp) metabolism is a major mechanism of immunomodulation and depletion of extracellular Trp has been shown to induce apoptosis of T cells [53]. Interestingly, the proteins involved in catabolism of Trp through the kynurenine metabolic pathway, such as IDO1 and KYNU, were enriched in M1 iPSDM (Figure 8A,C). In contrast, M1 polarisation-mediated Trp depletion also induced expression of tryptophanyl-tRNA synthetase WARS (Figure 8A,C), as shown previously [53].

Nucleotides are extracellular messengers actively secreted during cell stress and have immunomodulatory functions [54]. Our analyses showed that the enzymes involved in nucleotide metabolism, such as ADA, AMPD2, and CMPK, were differentially expressed in polarised iPSDM (Figure 8A,C). CMPK is a mitochondrial nucleoside kinase highly expressed in M1 M_Φ_ and has a role in inducing ROS production and inflammasome activation [55]. Interestingly, AMPD2 was found to be enriched in M2 iPSDM (Figure 8A,C). AMPD2 is an anti-inflammatory mediator that catalyses formation of IMP from AMP [56].

Furthermore, NOS2 is an enzyme that converts arginine to nitric oxide (NO) and citrulline and was found to be upregulated in M1 iPSDM (Figure 8A,C), as shown previously [57]. Interestingly, Ass1, an enzyme that recycles Arg from citrulline, was also highly expressed in M1 iPSDM (Figure 8A,C).

We observed that the rate-limiting enzyme in glycolysis, PFKFB2, was upregulated in M1 iPSDM (Figure 8A,C). In addition, we also observed enhanced expression of IRG1 on stimulation with IFNγ and LPS (Figure 8A,C), as shown previously [58]. IRG1 converts cis-aconitate (TCA cycle intermediate) to itaconic acid, which has been proposed to have an antimicrobial effect [58]. Interestingly, SHPK, a seduheptulose kinase was found to be upregulated in M2 iPSDM (Figure 8A,C). SHPK links glycolysis and the non-oxidative phase of the pentose phosphate pathway (PPP) and its overexpression has been shown to inhibit inflammatory cytokine production in M1 M_Φ_ [59].

Our findings suggest that there is a shift towards aerobic glycolysis and Trp metabolism in M1 M_Φ_, while M2 M_Φ_ rely on fatty acid oxidation for their survival and resolution of inflammation.

### 3.5. Differential Expression of Extracellular Mediators in Polarised iPSDM

**Figure 9:**
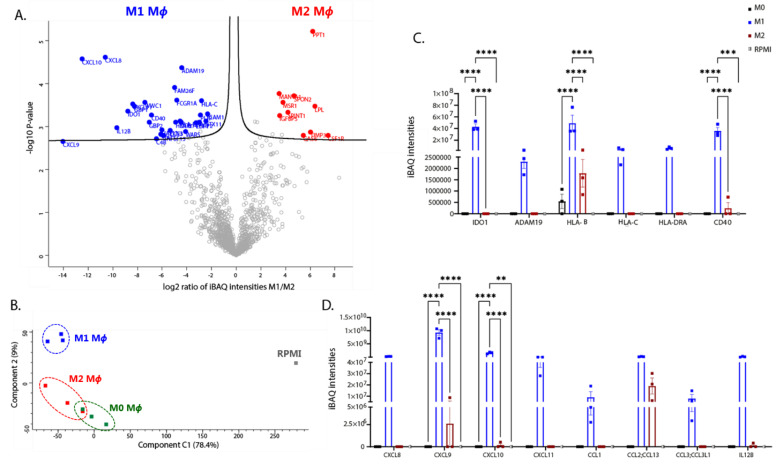
Secretome analysis of polarised iPSDM. (**A**) Volcano plot showing fold change changes in secreted proteins using log-transformed iBAQ intensities vs. log-transformed *p* values. (**B**) Principal component analysis plot showing clustering of the iPSDM secretome according to polarisation. (**C**,**D**) Histograms showing iBAQ intensities of cytokines/chemokines and other immune modulators. *p* values: ** 0.01, *** 0.001, **** 0.0001.

Polarisation induces release of soluble proteins such as cytokines and chemokines from M_Φ_, which orchestrate the immune response. Therefore, we characterised the secretion profile of polarised iPSDM. For this, a later time point (24 h post-polarisation) was chosen to allow sufficient time for polarisation-induced protein synthesis and secretion. The culture supernatants were concentrated, processed, and analysed by using LC-MS/MS. More than 1300 protein hits were identified that were reproducibly released from the polarised iPSDM. iBAQ intensities were used for analysing secretome data, as that normalises the MS intensity of each protein by the corresponding number of peptides detected. A volcano plot was generated by using the iBAQ intensities of M1 and M2 polarised iPSDM and the differentially secreted proteins were highlighted (Figure 9A and Table S2). PCA analyses of the secretome showed that the secretory profile of M0 and M2 iPSDM are more similar compared to the M1 iPSDM (Figure 9B), reminiscent of the cell-associated proteome profile. Furthermore, our proteome analyses (harvested 48 h post-stimulation) also revealed differential expression of cytokines, chemokines, and ECM proteins in polarised iPSDM (Figure S4).

Both proteome and secretome analyses detected C-X-C subfamily chemokines, such as CXCL8, CXCL9, CXCL10, and CXCL11 in M1 iPSDMs (Figure 9D and Figure S4), which is in line with previous studies [24,60]. Moreover, C-C motif chemokines, such as CCL1, CCL2, and CCL3, were also released specifically from M1 iPSDM (Figure 9D). As shown previously [24], polarisation of iPSDM with IFNγ and LPS increased secretion or expression of proinflammatory cytokines, such as IL12 (β-subunit) (Figure 9D) and IL1β (Figure S4).

Transmembrane glycoproteins, such as CD40, FCGR1, ICAM1, and HLA molecules were also found to be enriched in the secretome of M1 iPSDM (Figure 9C). Interestingly, CSF1R, the receptor for cytokines such as M-CSF and IL-34, was found to be released from M2 iPSDM (Figure 9A). IL-4 stimulation also induced secretion of the scavenger receptor, MSR1, from iPSDM (Figure 9A). These membrane proteins could perhaps be released by proteolytic cleavage or membrane shedding [61,62,63].

ADAMs are a family of metalloproteinases involved in ectodomain shedding of cell surface receptors, adhesion molecules, and cytokines. ADAM19 was specifically released from M1 iPSDM (Figure 9C). In addition, our proteome analyses showed that the expression of ADAM9 and ADAM10 was downregulated in M1 iPSDM (Figure S4). Interestingly, TIMP3, an inhibitor of metalloproteinases, was found to be enriched in M2 secretome (Figure 9A).

Consistent with our proteome analysis, metabolic enzymes such as IDO1 and WARS were released from M1 iPSDM (Figure 9A,C). In contrast, M2 polarisation induced secretion of enzymes involved in lipid metabolism, such as LPL and PPT1 (Figure 9A).

Interestingly, GAS6 was found to be released at high levels from M2 iPSDM (Figure 9A). GAS6 is a ligand for TAM receptors and has an important role in mediating efferocytosis by M_Φ_ [64].

In addition to the extracellular mediators reported in the literature, our secretome analyses identified a number of proteins released differentially from polarised M_Φ_.

## 4. Conclusions

This study used unbiased profiling of proteome and secretome changes in differentially polarised iPSC-derived M_Φ_ to obtain a better understanding of M_Φ_ functions in terms of cell surface phenotype, intracellular signalling, immune functions, and metabolic signatures. This study demonstrates that iPSC M_Φ_ are promising tools for understanding M_Φ_ biology, as they exhibit similar polarisation profiles and functions as monocyte-derived M_Φ_. We believe our comprehensive proteome and secretome data set will be a useful resource in the M_Φ_ field.

In addition to the established surface markers, our study identified novel markers in differentially polarised M_Φ_. The alterations in expression of cell surface proteins likely influence the functions of M_Φ_ in the way they respond to the microenvironment. Our analyses confirm that M1 M_Φ_ are specialised in pathogen defence by presenting antigens and priming T cells for activation, while M2 M_Φ_ are efficient at migrating to the sites of wound healing and mitigate inflammatory response by mediating efferocytosis of apoptotic cells. We also detected polarisation-induced changes in intracellular signalling, expression of transcription factors, and secretion of cytokines and chemokines. These phenotypic and functional changes in M_Φ_ were also accompanied by dramatic shifts in expression of enzymes and other proteins associated with cell metabolism. M1 M_Φ_ utilise aerobic glycolysis and breaks in Kreb’s cycle to produce microbicidal products, such as itaconate. Fatty acid oxidation is pronounced in M2 M_Φ_, resulting in production of mediators that resolve inflammation and promote wound healing, while upregulating the non-oxidative branch of PPP. Overall, this study provides new insights into how polarisation stimuli differentially regulate M_Φ_ immune function.

## Data Availability

Mass spectrometry proteomics data are deposited with the ProteomeXchange consortium via the PRIDE repository. “Proteomic analysis of macrophages derived from induced pluripotent stem cells”, accession number PXD029776. “Secretome analysis of polarised macrophages derived from induced pluripotent stem cells”, accession number PXD029831.

## Supplementary Materials

The following supporting information can be downloaded at: https://www.mdpi.com/article/10.3390/biomedicines10020239/s1, Tables S1 and S2: Showing copy numbers per cell data for differentially regulated proteome and secretome protein hits, respectively. Figure S1: Demonstrating expression of previously characterised surface markers CD80, CD86, MHC-II, and CD206 in polarised iPSDM. Figure S2: Showing expression of previously characterised surface markers CD80 and CD206 in polarised MDM. Figure S3: Expression of previously characterised (CD80, CD206) and potential novel surface markers (LILRB2, PILRA, CD109, and CD300a) in polarised MDM from two independent donors. Figure S4: Proteome analyses of cytokines, chemokines, and other secreted factors.
