# Supplementary figures and images for: Quantitative Proteomics of Polarised Macrophages Derived from Induced Pluripotent Stem Cells
Source: Biomedicines. 2022 Jan 23;10(2):239. doi: 10.3390/biomedicines10020239 (PMC8869710; doi:10.3390/biomedicines10020239)

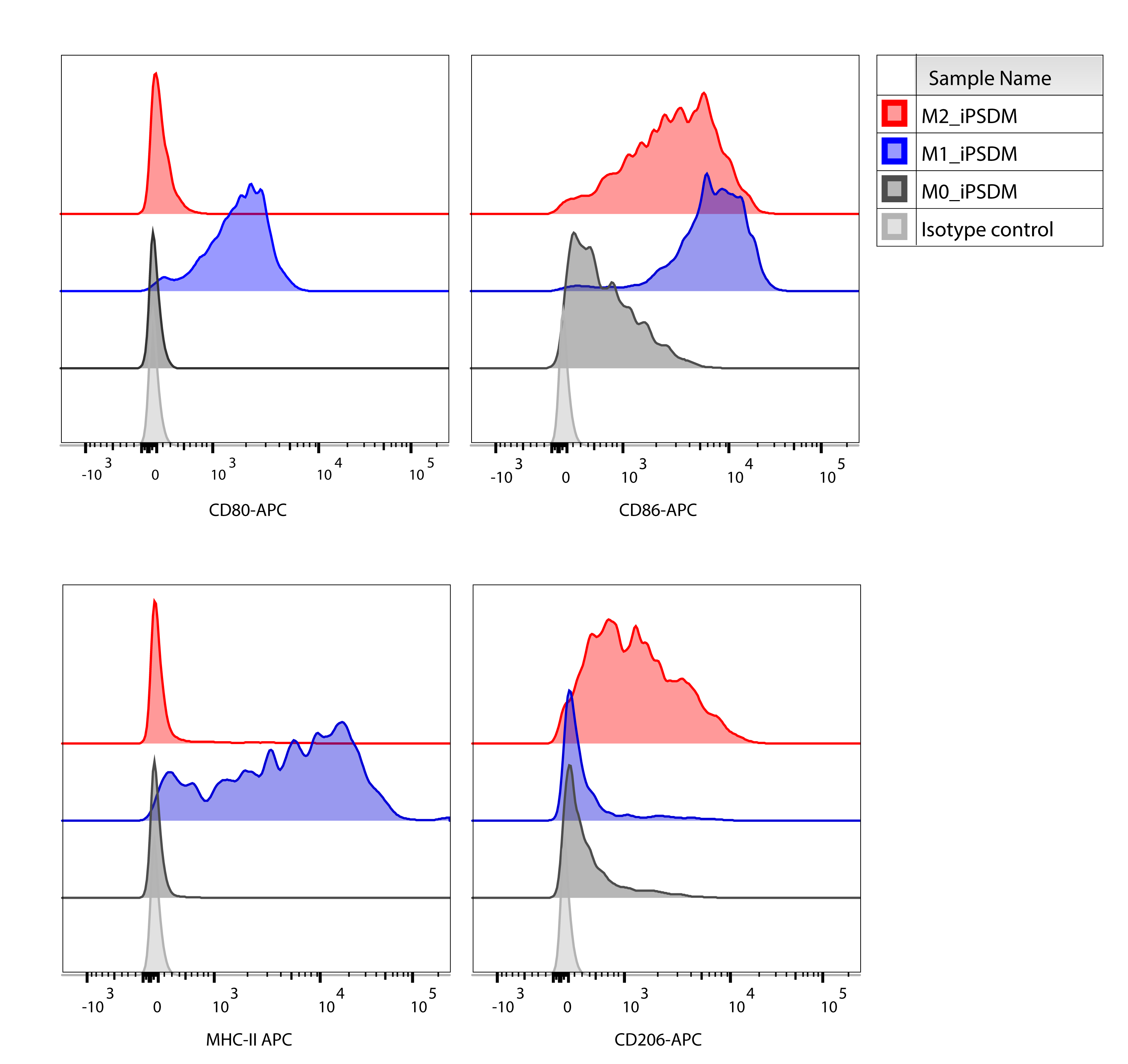

Supplement: Supplementary file 1 [file biomedicines-10-00239-s001.zip › biomedicines-1508353-si/Figure S1.tif]

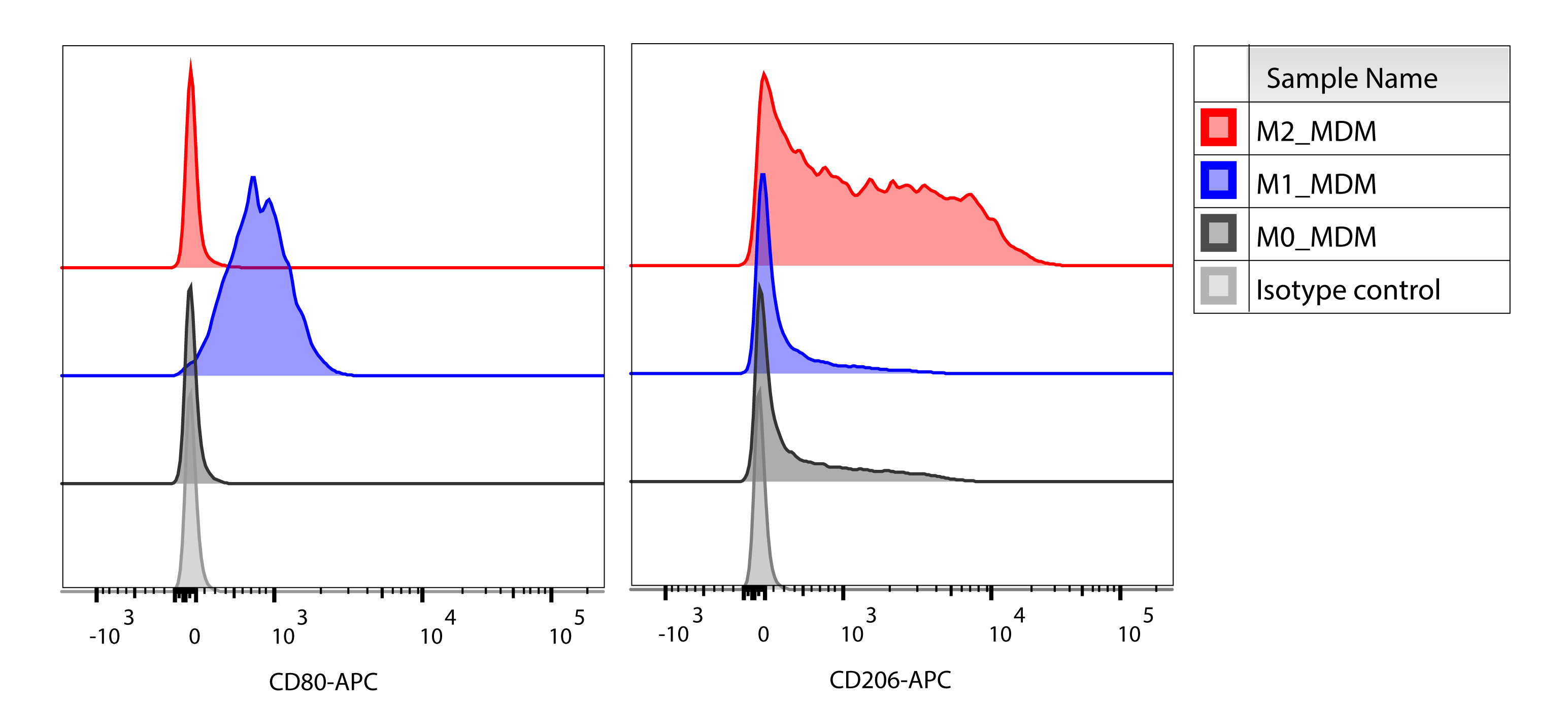

Supplement: Supplementary file 1 [file biomedicines-10-00239-s001.zip › biomedicines-1508353-si/Figure S2.tif]

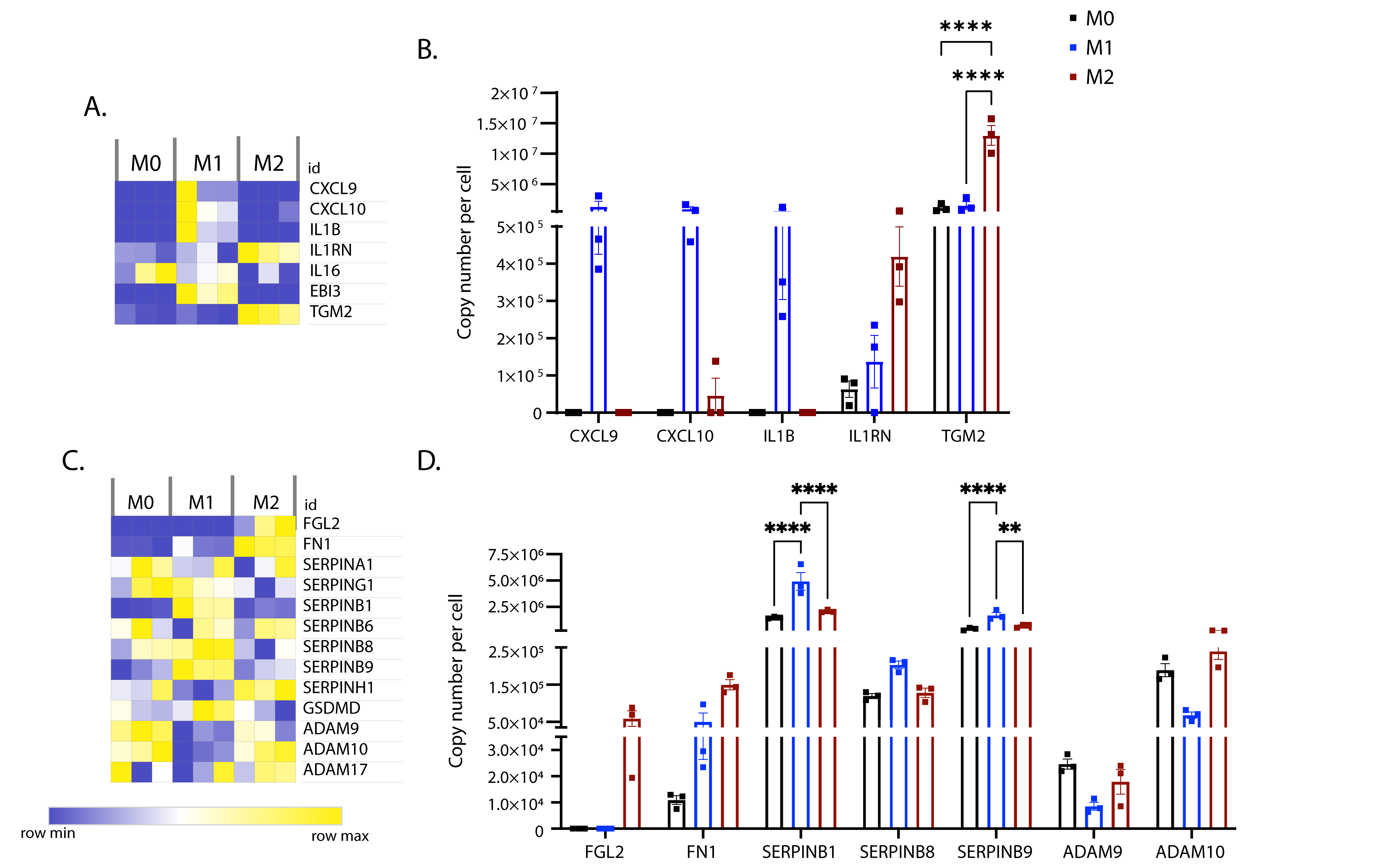

Supplement: Supplementary file 1 [file biomedicines-10-00239-s001.zip › biomedicines-1508353-si/Figure S4.tif]
